# Investigation of Redundant Visual Patterns to Improve Usability of Labels

**DOI:** 10.1177/00187208261429742

**Published:** 2026-03-19

**Authors:** Yuval Bitan, Abed El Hamid Fahoum, Greg Fukakusa, Cynthia Valdron, Paul Milgram

**Affiliations:** 1 108801 Ben-Gurion University of the Negev, Israel; 2 7938 University of Toronto, Canada

**Keywords:** labelling, visual search, visual patterns, display design

## Abstract

**Objective:**

To examine whether adding well designed visual patterns to labels can increase efficiency of searching for labelled items.

**Background:**

Errors of substitution can occur when selecting among similarly appearing items, especially under adverse conditions and time pressure.

**Method:**

Two consecutive studies investigated effects of adding visual patterns to simulated labels in a visual search task. Both studies attempted to objectively design distinctive patterns, based on normative constraints. Study 1 compared response times and accuracy for target items comprising Text+Patterns, Text-Only, or Pattern-Only, involving searching for target items (based on working memory recollection) within an array of distractors. Study 2 additionally addressed memorability and spatial frequency content of patterns.

**Results:**

Study 1 indicated longer response times for simulated labels without patterns. No significant effects were found due to spatial frequency of patterns, nor to Pattern-Only versus Text+Patterns. Study 2 also showed longer response times and more errors for non-patterned labels. Weaker evidence was found to support improved performance for lower effective spatial bandwidth content of patterns.

**Conclusions:**

Evidence was found for decreased response times, and possibly fewer errors, when searching for target labels containing redundant information using potentially familiar visual patterns. The need exists for objective tools for normatively designing such patterns, potentially based on spatial frequency content.

**Applications:**

In addition to label reading in general, medication labels may particularly benefit from adding patterned labels, to assist paramedics, pharmacists, nurses, anaesthesiologists, as well as lay consumers.

## Introduction

The present study is based on the accepted premise that if operationally critical objects can be designed such that they appear not only maximally salient but also *maximally distinguishable* from each other, the efficiency of visual search will likely be increased (Geng & Witkowski, 2019; Wickens et al., 2013). It is additionally postulated that errors of substitution when selecting among otherwise similarly appearing objects may be further reduced if the appearance of target objects can be designed also to be *maximally memorable*, in the sense that users will more likely possess a useful mental image prior to initiating visual search. Errors of substitution refer here to one’s correct decision about which object to select, followed nevertheless by incorrectly selecting a different object (Reason, 1990). For practical purposes, we focus here on *labelling*, as a primary pragmatic means of modifying object appearance.

The challenges underlying these seemingly reasonable premises, in addition to demonstrating their validity for specific practical applications, include issues such as how to actually design appropriate labels (subject to practical constraints), in conjunction with establishing effective methods for evaluating new label designs within the context of their eventual use. In this paper we report initial results from within a longer-term research programme to examine whether *appropriately designed visual patterns added redundantly to existing labels* might be able to provide the advantages outlined above. Although our research was originally motivated by the potential impact of improving the labelling of medications, the present paper is broader in its scope. That is, our focus here is on potential general advantages, in principle, of adding redundant patterns to existing labels, independent of the domain of application. Some portions of the present results have been reported in Fukakusa et al. (2018) and Fahoum and Bitan (2020).

### Illustration

**Figure 1.**
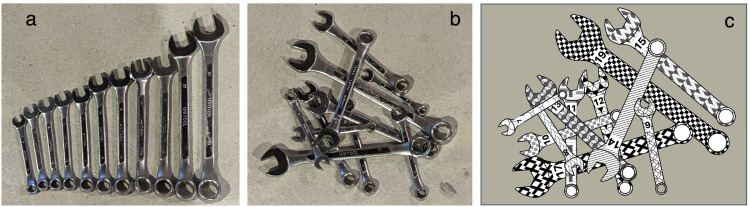
Hypothetical effect of adding patterns on wrench selection task. (a) Standard 11 piece wrench set, organised according to size. (b) Same wrench set, but scattered about on shop floor. (c) Simulated image of scattered wrench set, but with different pattern added to each wrench.

As a generic illustration, consider Figure 1, comprising three images, each showing a set of common wrenches of various sizes. The photo on the left depicts an organised arrangement, idealised for facilitating selection of the appropriate wrench for a particular task. The middle photo shows a more realistic situation, in which the same set of wrenches is scattered about. Except for experienced mechanics, selecting the appropriate tool in the middle case would likely require close examination, involving reading the size inscribed on each wrench and/or possibly involving trial-and-error. Note that this example is in fact a classic *absolute judgement task,* well-known in the human factors literature to be subject to the ‘7±2’ constraint (Miller, 1956).

The image on the right of Figure 1 depicts the contrived case for which the appearance of each wrench has been modified by superposing a different distinctive visual pattern – essentially creating an additional information display channel. The premise of the present paper is that even non-expert users, after having become familiar through repeated use of the patterned wrenches, are likely to locate and select any particular wrench more quickly and/or with fewer errors relative to the arrangement shown in the middle photo – that is, without having to rely solely on expressly reading wrench sizes.

### Factors Affecting Effective Labelling

In the above illustration superposed patterns have been used to modify the entire appearance of each wrench. For many practical cases, however, one is more likely to be constrained to modifying existing labels. In order for such modifications to be effective two important factors should be considered:

- *Distinguishability*: when two or more objects differ sufficiently in physical appearance, errors of substitution are less likely to occur.
- *Memorability*: when the appearance of an object is easy to recall, this may facilitate the search process, leading more rapidly to correct selection.

As illustration, consider the case of an experienced paramedic treating a patient. She knows which medication (and dosage) she wants and clearly, depending on the state of her patient, she wants to obtain that medication as quickly as possible. A typical procedure would entail reaching into her medication case, searching for the intended medication and, before administering it, reading the label to confirm the veracity of both the medication name and dosage. To efficiently locate the desired medication among many others in her bag, it should be helpful for the paramedic to know in advance what the particular medication looks like – hence the relevance of *memorability*. It is also reasonable to presume that finding the target medication might occur more quickly, and/or more accurately, if the medication were less likely to be confused with other medications – hence the relevance of *distinguishability*.

To facilitate such tasks, we are proposing to add *visual patterns,* as a potential means of increasing the number of *redundant visually identifying characteristics* within an ensemble of labels, with the goal of reducing both search times and frequency of errors-of-substitution, prior to the final label verification step. In the context of the present paper, we use the term ‘modified label’ to refer to all label-plus-unique-added-pattern combinations.

## Related Labelling Research

Much of the relevant research has focussed on labelling of medications, where one presumably obvious way to reduce errors of substitution (other than relying exclusively on diligence while reading labels) is to redundantly provide identifying colours to labels (Merry & Webster, 1996). In one laboratory simulation study of an intensive care unit (Porat et al., 2009), it was shown that the use of new colour-coded labels improved identification of IV bags, reduced performance time for overall tasks, and improved identification of errors at the treatment setting. Another recent study on the use of colour coded compartmentalised syringe trays also showed the advantage of colour-coded labels (Laxton et al., 2024).

In contrast however, another 36-month study within an anaesthesiology department found no reduction in syringe swap errors due to the introduction of colour-coded labels (Fasting & Gisvold, 2000). Others have found shape and size rather to be the greatest predictors of swap occurrences, regardless of label colouring (Bitan et al., 2024). Some researchers have suggested that colour coding might even contribute to errors, by virtue of the possibility that reliance on colour might create a false sense of security, eventually resulting in increased neglect of label reading (Nunn & Baird, 1996).

With regards to accepted practice, the Institute for Safe Medical Practices has urged that colour be used as part of a systematic solution for preventing medication errors, but not as the *entire* solution. Other attempts to address this problem have focussed on the *formatting* of labels, in terms of shape, font size, border width, letter spacing, line width, and so forth (Silver & Braun, 1993), in addition to some studies examining the efficacy of ‘Tall Man’ lettering (Darker et al., 2011). Much of that research has focussed, however, on the *readability* rather than the *distinguishability* of medications. Another important consideration is the potential for confusion among actual ‘look-alike, sound-alike’ names of medications.

## Designing Suitable Visual Patterns

Our original inclination for testing the proposed concept was to design a visual search task and compare performance for targets with and without added patterns. In addition to the challenge of designing an appropriate task, however, we realised that such an experiment would likely have little impact unless we were also able to devise an objective method for *designing* suitable visual patterns, for which our two primary criteria were *maximal distinguishability* and *memorability*. The second (arguably more difficult) criterion is based on the supposition that minimal effort should be required to learn the patterns, ideally implicitly through day-to-day use. In this section, we describe our efforts at pattern designing.

### Designing for Distinctiveness

Much of the visual search literature provides insights into perceptual factors that facilitate detection of targets within a background of distractors. Some of those factors include object colour and shape (Buetti et al., 2019), as well as number of objects in the search field, defining characteristics of targets, similarity between distractors, and similarity between target and distractors (Wickens et al., 2013). In general, search performance would likely improve if, rather than examining objects serially, one were able to search objects within an array in parallel. Certain features may further be more likely to be processed pre-attentively if perceived to be more distinctive (Treisman & Gelade, 1980).

According to guided search theory (Wolfe, 2021), it should be possible when designing patterns to systematically employ well-defined sources of pre-attentive information that guide observers to attend to particular features in parallel while ignoring others, and thus identify an intended target quickly and reliably. In particular, Wolfe has proposed five explicit sources of pre-attentive information: top-down and bottom-up feature guidance, priming, reward, and syntax/semantics. For example, if a target containing diagonal lines were sought, an observer could be guided to perceive these as distinct from neighbouring patterns that do not contain diagonal lines, thus leading to more rapid detection.

One clear difficulty with existing theories of pre-attentive processing, however, is that most related studies have typically focussed on identifying unique targets within a sea of distractors that are different from the targets but similar to each other. In the present case, our challenge is to design *all* of the ‘distractors’ to be maximally distinct *also from each other*. In that regard, it is nevertheless important to consider whether Wolfe’s five sources of pre-attentive information, although seemingly useful for the present goal of designing additive patterns, would in fact be viably sufficient for designing *large numbers of distinctive patterns* that would likely be necessary for practical applications.

### Quantifying Features of Patterns

Much of our effort was based on the supposition that if it were possible to define an objective metric that quantitatively reflects critical distinctive properties of a pattern, a design tool could be developed based on selecting candidate patterns that are broadly distributed across the spectrum of values of that metric. Some research relevant to this goal has been done on perception of *textures*. Although patterns are not the same as textures, which refer primarily to the physical quality of surfaces, some of the classic work by Julesz (1981) on textures has added to our understanding of how humans can perceive groups of features that combine to form patterns.

One approach to texture classification was that of Tamura et al. (1978), who had participants rank an assortment of Brodatz (1966) textures along six dimensions: coarseness, contrast, directionality, line-likeness, regularity, and roughness, the results of which were compared to a computational model based on those parameters. In an analogous study, Amadasun and King (1989) compared their computational model to rankings based on five parameters: coarseness, contrast, busyness, complexity, and textural strength.

Whereas participant responses in both of those studies were based on predefined texture dimensions, Rao and Lohse (1993, 1996) instead applied multidimensional scaling to the results of a sorting experiment, again using Brodatz images, to identify three orthogonal dimensions of texture: repetitive versus non-repetitive; high-contrast/non-directional versus low-contrast/directional; and granular/coarse/low-complexity versus non-granular/fine/high-complexity. Collectively those studies provided guidelines for both defining distinctive patterns and quantifying them along orthogonal dimensions.

### Spatial Frequency

An additional approach we took to quantifying distinctiveness properties of patterns was based on studies showing that much of human perception involves decomposition of visual scenes according to spatial frequency components (Kauffmann et al., 2014). Because any pattern necessarily comprises some aspect of spatially cyclic repetition, we hypothesised that a metric based on quantifying the *spatial frequency* properties of individual patterns should be useful for designing for *distinctiveness* of those patterns. For clarification, spatial frequency is defined as “the number of pattern cycles per unit angle subtended by the eye” (Ware & Knight, 1995). Because any real-world physical image in fact comprises an infinite number of frequencies, our challenge, as outlined below, was to define a metric that reflects the *dominant* spatial frequencies contained within any given pattern.

Two important constraints for designing useful patterns are that their dominant spatial frequencies be neither too low nor too high. For the former case, the possibility exists that for a given label size the number of cycles of a pattern might be too few for it to be perceived as an actual pattern. If dominant spatial frequencies are very high, on the other hand, there would consequently be more details per unit visual angle, which in turn would require the viewer to resolve very dense pattern details. Assuming some range of suitable intermediate frequencies, our premise was therefore that images with relatively high spatial frequency content, containing more details, will be harder to distinguish among a mix of patterns, whereas images with predominantly low (but not too low) spatial frequency components will appear more regular and thus easier to identify and memorise.

In all cases, to ensure perceptual acuity, the above criteria require that patterns explicitly take into account both the physical dimensions of the labels upon which they will be placed and the anticipated distance at which they will be viewed, based on the following relationship:

(1) $$Visual\;angle\;\left(in\;degrees\right)=2\;\tan^{-1}\left(\frac{Visual\;Object\;Size/2}{Viewing\;Distance}\right)\frac{180}{\pi }$$

### Other Constraints

For practical reasons, we also stipulated that useful patterns be independent of rotation. For example, it would not be logical to employ one pattern comprising vertical stripes and another comprising horizontal stripes, for the simple reason that one could never be sure of an object’s orientation (e.g., a medication within a paramedic bag).

Another important decision was not to rely on colour coding for our experiment. Although the use of colours clearly holds potential for adding redundancy to labels, as reviewed above, we reasoned that if there is indeed an advantage to be gained from adding patterns to labels, it would be more convincing to demonstrate this by using black-and-white patterns, rather than risk confounding our results due to potential interactions resulting from addition of colour.

### Practical Design Guidelines

Unfortunately, few of the publications reviewed provided guidelines for actually designing distinctive patterns. One notable exception is by Ware and Knight (1995), who developed texture producing algorithms based on orientation, size, and contrast, together with texton concepts proposed by Julescz (1981). Healey and Enns (1998) extended this concept by generating textural elements that varied in height, density, and regularity. Their test for visualising multidimensional datasets revealed that effectiveness depended on both target dimension and composition of the surrounding display. Most relevant was the work of Kujala and Lukka (2003), who used an algorithmic approach similar to that of Ware and Knight to generate patterns, with the added criterion that patterns be relatively simple, easily distinguishable, and recognisable. Their test for effectiveness for recognising and identifying hyperlinked documents indicated better identification performance for textured pattern backgrounds. Although somewhat limited in scope for our purposes, their results supported the potential advantages of adding patterned labels as hypothesised in the present research.

## Study 1

### Design of Experiment

To be relevant to real-world applications, an ideal visual search experiment should be carried out over an extended period of time, to allow participants to become acquainted with a set of distinctive and memorable patterns, preferably through repeated exposure, as part of some practical task. Due to practical constraints on our initial test of the proposed concept, however, we instead designed an experiment in which participants were presented with only a brief exposure to each particular pattern, which they were then asked to locate within a relatively large array of distractor patterns. Our reasoning was that if detection performance could be enhanced following even brief exposure to individual target patterns, using only working memory, this would support our premise that performance would likely be further improved if users were to become well acquainted with those patterns and thus exploit their long-term memory. Our first experiment therefore focussed on distinctiveness of patterns, while setting aside the other important factor of memorability.

**Figure 2.**
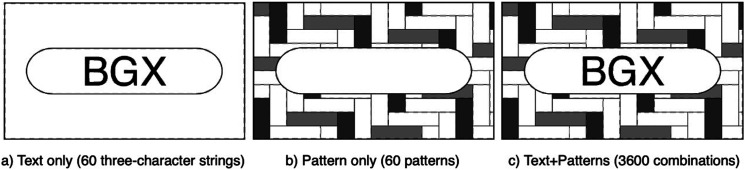
Examples of (a) Text-Only, (b) Pattern-Only, and (c) Text+Pattern stimuli.

**Figure 3.**
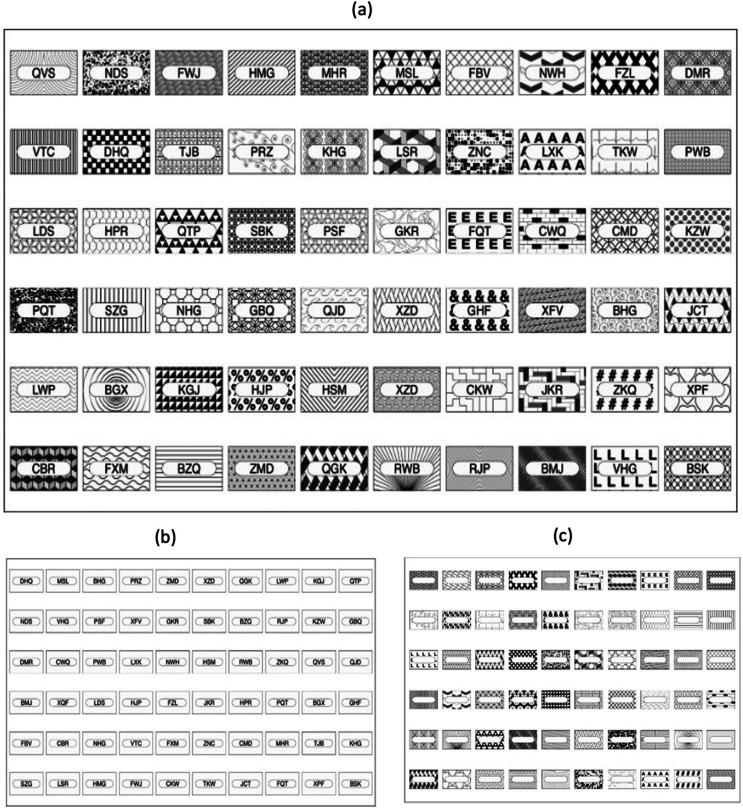
Examples of 6×10 arrays of stimuli used in experiment: (a) Text+Pattern stimuli, (b) Text-Only stimuli, and (c) Pattern-Only stimuli. (Images (b) and (c) are presented for illustrative purposes, and thus have been minimised.)

The experiment (with full details provided in Fukakusa, 2018) was within-subjects, comprising 60 different stimuli, blocked according to three conditions: {Text-Only; Pattern-Only; Text+Pattern}, as illustrated in Figure 2. The procedure, which was analogous to that of Irwin et al. (2013), involved briefly presenting a target stimulus on a screen, after which an array comprising 60 stimuli from the same condition appeared immediately, as illustrated in Figure 3. Participants were asked to find the original target stimulus, which was always present within the array, as quickly as possible. The dependent variables were response times (from onset of stimulus array to response) and detection accuracy.

### Design of Stimuli

The reason for presenting 60 stimuli was to make the task sufficiently challenging and thus likely to produce different results across the three conditions. By presenting tens of different alternatives, we also endeavoured to simulate, as a practical example, the number of different medications that might be found in a paramedic bag.

The Text-Only stimuli were designed with the goal that that condition would be neither too difficult nor too easy. In other words, it would have been simple to design an ‘unfair’ experiment comprising very difficult-to-recall text targets, for which performance on conditions comprising detecting patterns would likely have excelled. Conversely, we wanted to avoid highly recognisable text stimuli (e.g., ‘cat’ or ‘man’) for which the use of additional patterns would not likely be useful. Following extensive pilot testing we converged on three-character strings of random consonants for text stimuli, produced using the string generating tool at https://www.random.org/.

As explained above, the patterns used should ideally be maximally distinctive, in support of rapid top-down visual search. Together with that criterion, we also aspired to generate patterns that were hypothetically distributed along a set of quantifiable dimensions of coarseness, contrast, and complexity, as defined using the Amadasun and King (1989) algorithms.

For each pattern we also computed an estimate of the dominant spatial frequency, using the 2D Discrete Fourier Transform (DFT) function using the Matlab formula:

(2) $$I\left(f_{x},f_{y}\right)=\overset{M-1}{\underset{x=0}{\sum }}\overset{N-1}{\underset{y=0}{\sum }}i\left(x,y\right)e^{-j2\pi \left(f_{x}x+f_{y}y\right)}$$

where i(x,y) represents the M×N array of pixel values of a particular image. The resulting transform comprised a 2D array of intensities, I(f_x_,f_y_), for each pair of spectral frequency coordinates {f_x_,f_y_}. For each transformed function, Matlab provided a value that we used as a metric representing a first approximation to the dominant spatial frequency (in cycles/degree) for that pattern, based simply on the frequency (either f_x_* or f_y_*) corresponding to the maximum magnitude component I(f_x_*,f_y_*).

The absence of well-defined prescriptive recipes for concurrently applying the four not necessarily orthogonal parameters being considered {courseness, contrast, complexity, spatial frequency} impeded their direct use for designing patterns for the present experiment. We were nevertheless able to use these parameters as guidelines for improvising patterns based on such primitives as alphabetic characters, various shadings, and fill patterns, as illustrated in Figure 3. In all cases details within the patterns were also subjected to limitations of stimulus size and viewing distance, as explained in equation (1).

### Procedure

The experiment was run using OpenSesame 3.1.8 (Mathôt et al., 2012) on a MacBook_Pro running OS_10.13.3, and a 27” BENQ external monitor with resolution 2560×1440 pixels. Participants sat at an unconstrained distance of 63 cm from the screen, which resulted in each stimulus (of size 232×132 pixels) subtending visual angles of 4.9° horizontally and 2.8° vertically. The 63 cm distance was chosen so that the dominant spatial frequencies of the patterns would lie within the range of 1–10 cycles/degree, to ensure that pattern details would likely be discernible (Owsley et al., 1983).

Each trial began with a 2s presentation of a randomised target stimulus, which was then replaced by a randomly arranged array of 60 stimuli of the same condition (one of which was always the target), each of the same size (232×132px) as the original target presented. Participants were asked to locate and click on the target within the array as quickly as possible, with no time restriction and without feedback. Each participant underwent 190 trials (10 practice trials plus 60 trials for each of 3 stimulus conditions) of a within-subjects design. Due to the randomisation procedure, it was possible that some pattern and text conditions occurred more than once.

### Participants

Thirty participants aged 22-40 (14 males, 16 females) were recruited, all with normal or corrected-to-normal vision. Each participant provided informed consent and was compensated $15 for approximately 1 hour of their time. The experiment complied with the APA Code of Ethics, and was approved by the University of Toronto Research Ethics Board (Reference #34985).

### Hypotheses

Our hypotheses about task performance were based on the general supposition that the presence of visual patterns, especially those with lower dominant spatial frequencies, would reduce response times and would also reduce error frequency when accompanied by text. In particular:

**H1a**: Response times (RTs) for both Pattern-Only and Text+Pattern conditions were expected to be smaller than for Text-Only, due to increased visual distinctiveness of the patterns.
**H1b**: RTs for Text+Pattern were expected to be equal to or greater than for Pattern-Only, due to the potential for extra processing needed for verifying the patterns by also reading the text (with rejection of H1b indicating dominance of text over pattern cues).
**H2a**: The Text+Pattern condition was expected to generate the fewest errors, due to the redundancy of the two information channels.
**H2b**: The most errors were expected for the Pattern-Only conditions, due to participants’ relative unfamiliarity with the patterns, following the brief exposure.
**H3a, H3b**: Both RTs and error rates were expected to be larger for pattern stimuli comprising higher dominant spatial frequencies, due to smaller distances separating details in the patterns, as explained in our discussion about designing suitable visual patterns.

### Results and Analysis

**Figure 4.**
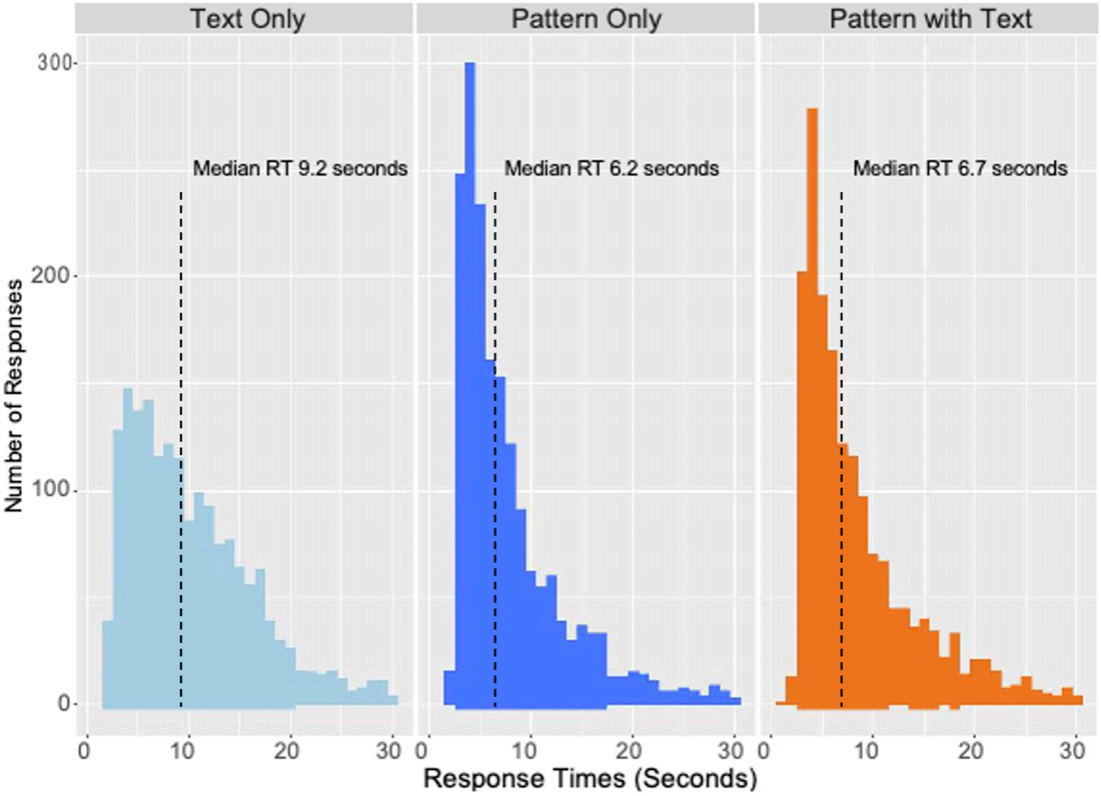
Histograms of response times (RTs) for the three conditions.

Figure 4 shows distributions of collective RTs for each of the three conditions, together with the median values. Due to inability to normalise the data, they were analysed non-parametrically in R using Friedman non-parametric repeated-measures (Field et al., 2012), resulting in a significant effect due to conditions $\left(\chi^{2}\left(2\right)=27.5,p<.001\right)$ . Consistent with Hypothesis H1a, post-hoc Wilcoxon signed-rank tests with Bonferroni corrections revealed that Text-Only RTs (median = 9.2s) were significantly greater than both Pattern-Only (median = 6.2s) and Text+Pattern (median = 6.7s), (r = −.09, *p* < .001 and r = −.07, *p* < .001 respectively). Because no significant differences between Pattern-Only and Text+Pattern were found, however, Hypothesis H1b was not supported.

Error rates were low across the experiment as a whole (120 errors out of 5324 trials, or 2.3%), resulting in no significant differences among the three conditions. Neither Hypothesis 2a nor 2b was thus supported.

**Figure 5.**
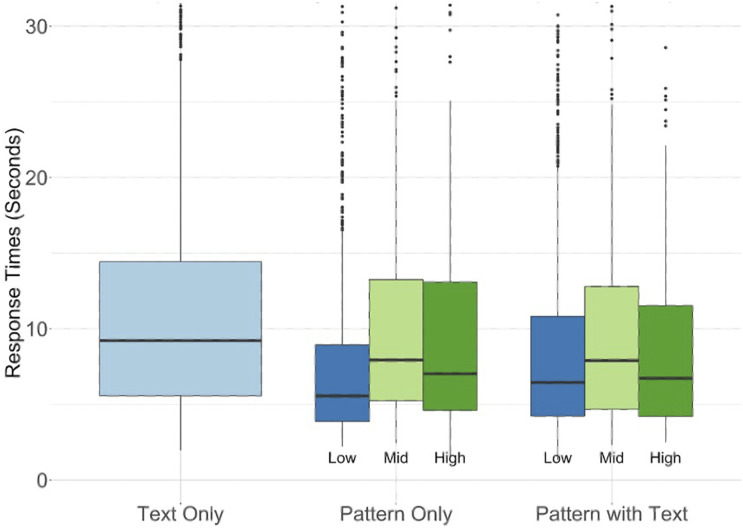
Response times versus conditions and frequency groups. Patterns subdivided into low, medium, and high spatial frequency regions.

Our estimates of the dominant spatial frequency for each pattern (equation (2)) ranged from 0.2 to 13.5 cycles/degree. To explore potential effects of this variable, we divided the patterns into three equal frequency ranges: {Low: 0.2–4.3; Medium: 4.4–8.7; High: 8.8–13.5}. Friedman non-parametric repeated-measures indicated a significant difference in response times, $\left(\chi^{2}\left(3\right)=32.5,p<.001\right)$ , as seen in Figure 5. Post-hoc test results, summarised in Table 1, once again supported Hypothesis H1a (for Low and High frequencies); however, only the Low–Mid frequency comparison supported Hypothesis 3a, which predicted greater response times for higher dominant spatial frequencies. Finally, due to the paucity of error data, no significant results were found to support of Hypothesis H3b′s prediction of more errors for higher spatial frequencies.

**Table 1.** Significant post-hoc tests for response times, for dominant spatial frequency ranges.

	Text-Only (Median = 9.2s)	Low Frequency (Median = 6.0s)	Mid Frequency (Median = 8.0s)	High Frequency (Median = 7.0s)
Text-Only		***p < .001, r = −.08		***p < .001, r = −.07
Low frequency			***p < .001; r = −.07	

### Takeaways from Experiment 1

Our first attempt to test the concept of facilitating visual search by providing additional patterns was provisionally supported by the shorter RTs for both the Pattern-Only and Text+Pattern conditions relative to Text-Only. Although the Pattern-Only search times were not shorter than Text+Pattern, they were also not longer, and the combining of the two modalities also did not appear to introduce additional errors. The shorter RTs for low–dominant relative to medium–dominant frequency patterns provided only slight support for the potential to use frequency content for designing maximally distinctive patterns.

## Study 2

### Design of Experiment

Our follow-up study followed the same general procedure as the first study, for consistency, but went further in terms of: (a) partially addressing the issue of memorability, (b) more closely simulating a realistic label search task, and (c) improving on our initial examination of spatial frequency (explained in detail below).

### Design of Stimuli

As a small step towards increased realism, instead of random consonants for the text stimuli as in Experiment 1, we used 30 names of fruits, vegetables, and animals (Table 2). These words, comprising 4–7 letters and 2–3 syllables, were selected using the English Lexicon Project (https://elexicon.wustl.edu/), and each had a log-HAL word frequency of 6.5–10 (Balota et al., 2007).

**Table 2.** Text Words Used in Experiment.

Orange	Lemon	Carrot	Coconut	Onion	Turtle	Gorilla	Eagle	Tiger	Leopard
Chicken	Melon	Garlic	Tomato	Dolphin	Cherry	Camel	Donkey	Mango	Rabbit
Koala	Pepper	Panda	Monkey	Banana	Potato	Zebra	Kiwi	Penguin	Peanut

**Figure 6.**
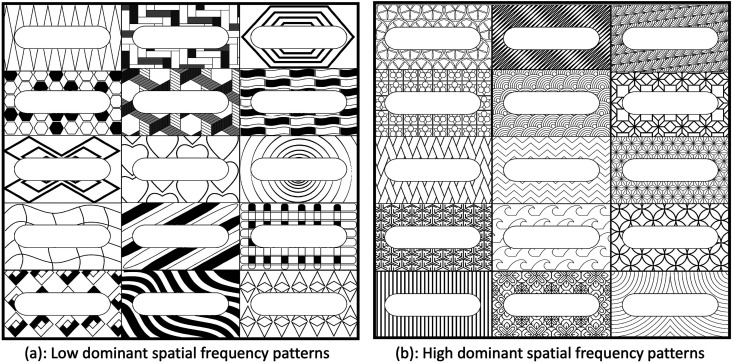
Examples of selected background patterns underlying (empty) oval text boxes.

Each stimulus, of size 320×200 pixels, contained a word printed within an oval, against a background that was either white or patterned. Two sets of 30 background patterns were designed, comprising predominantly low- and high-spatial frequencies, respectively. Figure 6 provides examples selected from each of the two background pattern sets.

In contrast to Study 1, the second experiment was conducted *between subjects*, distributed across three conditions, each comprising 30 stimuli with text superimposed on one of the following backgrounds:

- White background Labels (WL) (analogous to Figure 2(a)).
- Low Spatial Frequency Patterns (LSFP).
- Mixed Patterns (MP).

**Figure 7.**
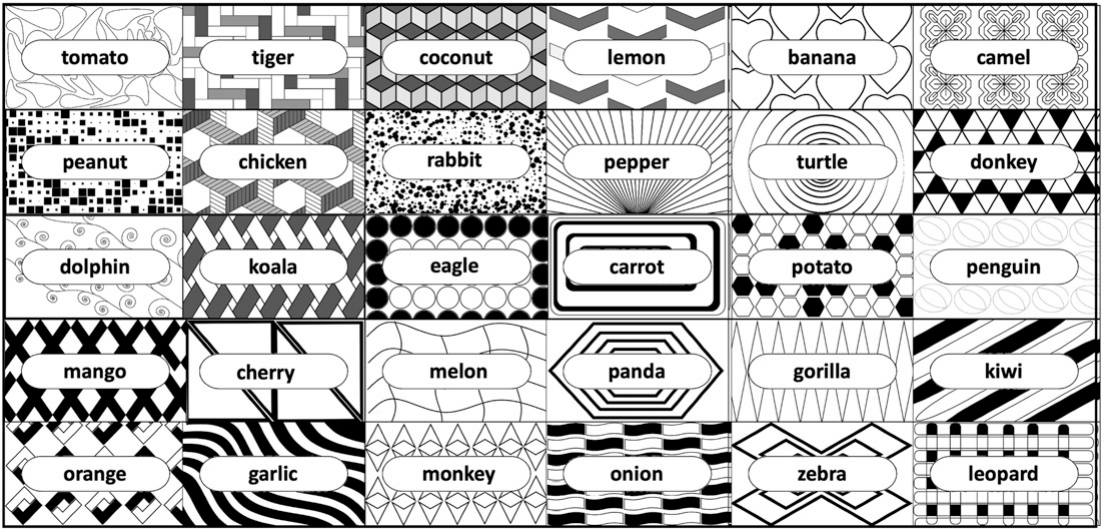
Example of array presented for low spatial frequency pattern (LSFP) condition.

The MP condition comprised a combination of 15 stimuli selected from the LSFP set and 15 stimuli with higher spatial frequency patterns. As a consequence, the 15 low spatial frequency patterns used in the MPL condition appeared also in the LSFP condition. All stimuli were randomly arranged within 6×5 arrays, as demonstrated by the typical LSFP condition array shown in Figure 7.

In one sense it would be correct to presume that the range of patterns used in the MP condition were analogous to those used in Study 1, in the sense that both experiments endeavoured to present participants with a wide range of spatial frequencies. An important difference between the two studies, however, was our addition of the separate LSFP condition, with the goal of more closely examining the potential advantages of lower spatial frequencies.

It is important to contrast the different randomisation strategies used in Studies 1 and 2. In the Text+Pattern condition of Study 1, each pattern was randomly matched with a different one of the text labels for *each* trial. In Study 2 on the other hand, although the pattern-plus-text combinations for the LSFP and MP conditions were generated randomly for each participant, in order to support learning of the stimuli the *same* pairings were maintained throughout the experiment. In all studies the *locations* of the various stimuli were randomly distributed anew for each trial.

### Procedure

Whereas in Study 1 participants were forced to rely on only working memory while seeking targets presented to them briefly prior to searching, in the second study a preliminary experimental phase was introduced, to give participants an opportunity in advance to first become acquainted with stimuli assigned to that participant. As a rudimentary test of whether learning actually occurred during this phase, participants were called back to a later session for the main target search portion of the experiment.

The preliminary phase was carried out in two stages. The first stage comprised a memory matching game in which participants were shown an array of 30 randomly arranged face-down stimuli, comprising 15 identical pairs whose format corresponded to their assigned experiment condition group. All participants within a group encountered the same texts and patterns, but each participant had different pairings of the two. By clicking successively on two stimuli at a time and remembering the stimuli and their locations as they were revealed, participants continued until they successfully identified all 15 identical pairs. Participants typically completed each memory game within 5–7 minutes.

**Figure 8.**
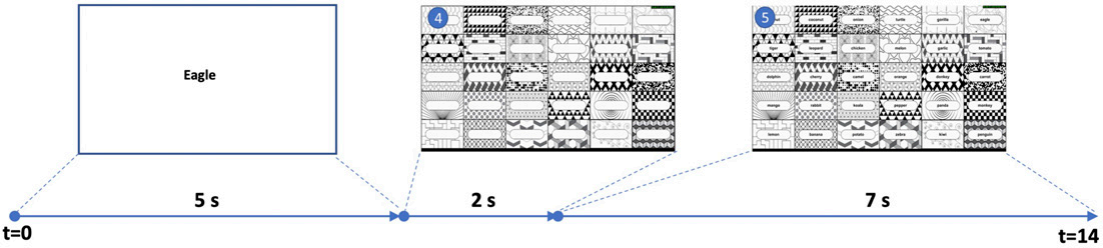
Time course of second stage of the preliminary phase. A target word was presented for 5s, followed by an array of patterns only (without text) for 2s, followed by the search task to find the target word, within 7s. No feedback was provided.

The second stage of the preliminary phase, which took place 1–2 days later, was designed to further strengthen the relationship between text content and associated patterns, as illustrated in Figure 8. Participants were first presented a single word on the screen for 5s and asked to remember it. They were then shown a 6×5 array of the 30 stimulus patterns for 2s, after which the text content appeared simultaneously within all the associated ovals for 7s. During that time participants had to find and identify the stimulus associated with the original target text by clicking on it as quickly as possible. Any presentation not responded to within 7s was considered as ‘missing’, and the control software automatically proceeded to present the next stimulus, without recording or replacing the missed event.

The procedure was repeated for all 30 stimuli, in randomised order. Note that, for consistency, during the 2s portion for the White Labels condition participants were shown an array of 30 white empty boxes, which were then filled with text for the last (7s) portion. This stage of the experiment took 7–9 minutes to complete.

**Figure 9.**
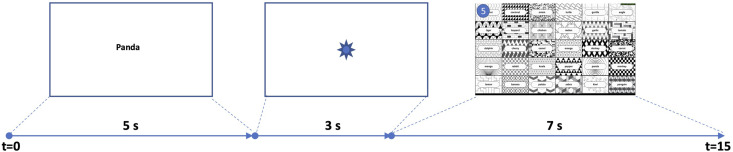
Time course of second phase of experiment. A target word was presented for 5s, followed by a fixation point for 3s, followed by the search task to find the target word within 7s. No feedback was provided.

The second phase of the experiment, using the same set of stimuli, was conducted not sooner than two days after the end of the first phase. The procedure, illustrated in Figure 9, was similar to that of Figure 8, except that, instead of viewing an array of text-free patterns during the intermediate period, participants were shown only a fixation point, for 3s, prior to the visual search task.

Consistent with our assertion that real-world users will in practice become acquainted with (memorable) patterns implicitly, simply through exposure to them, the decision was made not to provide explicit feedback to participants in any of our experiments.

Each participant underwent 30 trials; the entire experiment took 5–7 min to complete. As with Study 1, the dependent variables were response time and target detection accuracy. Any presentation not responded to within 7s was considered as ‘missing’.

Although carried out in a different lab, all equipment used for the second stage of the preliminary phase and the second stage of the experiment was functionally identical to that in Study 1. Presentations were managed with OpenSesame software, the 27” screen had identical 2560×1440 resolution, and participants were seated 63 cm away.

Referring to Figure 9, one might surmise that to successfully locate the target word during the final 7s, based on presentation of the target word during the initial 5s, *participants should theoretically have had no need for the surrounding patterns*, especially given that each target word appeared only once. On the other hand, *any improvement in performance for either of the pattern conditions, relative to the white label (WL) control condition, would arguably suggest the presence of some recollection about word-pattern pairings from the earlier preliminary phase*.

### Effective Spatial Frequency Bandwidth (ESFB)

For our analysis in Study 1, we defined a *dominant spatial frequency* metric based simply on the greatest local maximum |I(f_x_,f_y_)| value obtained from each DFT (equation (2)). One other goal of Study 2, however, was to better explore the potential influence of spatial frequency content by using a metric that encompasses a range, or *bandwidth,* of frequencies, rather than one single frequency.

**Figure 10.**
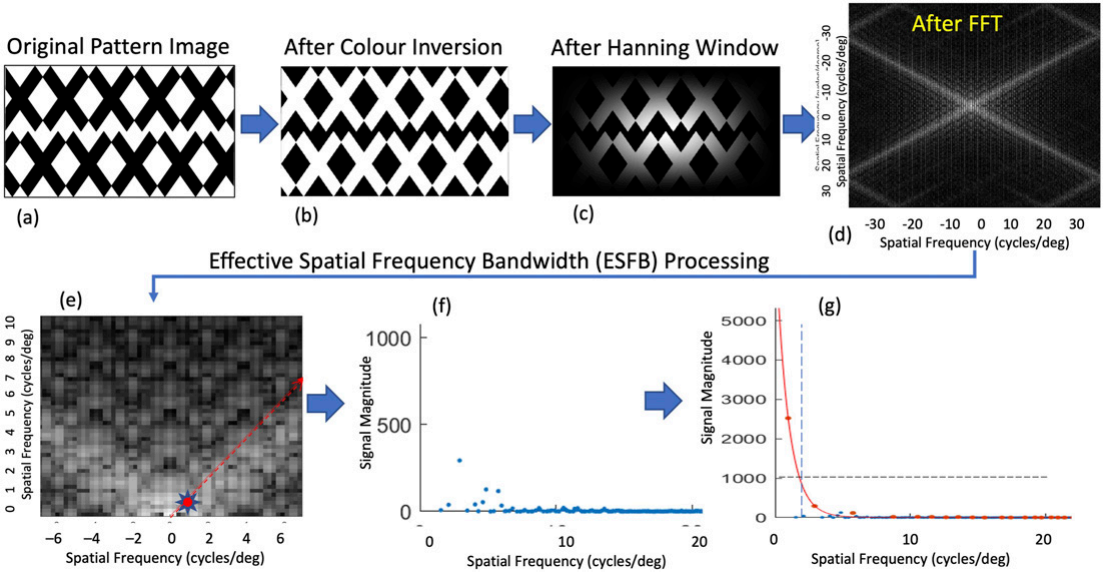
Example of process used to estimate effective spatial frequency bandwidth (ESFB). (a) Sample pattern. (b) After colour inversion. (c) After application of 2D Hanning Window. (d) After 2D Fast Fourier Transformation, shifting of frequency axes, and conversion of frequency units. (e) Magnification of same transformed image, showing only quadrants 1 and 2 (due to diagonal symmetry). Enlarged (red) dot depicts dominant frequency component (≈ 0.98 cycles/deg), following polar coordinate transformation. (f) (Blue) dots show successive values of peak magnitudes along the (red) polar ray indicated in (e). (g) Model fit of peak values shown in (f). (Note different vertical scales in (f) and (g).) Horizontal and vertical dashed lines depict derivation of ESFB value (≈ 1.82 cycles/deg), based on threshold magnitude = 1000 criterion.

Our procedure for deriving this metric is depicted through the example shown in Figure 10. For each pattern image we first inverted the pixel colour values, to more closely approach perception of images such as those comprising black pixels on a white background. Using Matlab, we then applied a Hanning window to the resulting image, to reduce biases arising from edge effects introduced by the DFT. This was followed by application of the *fft2()* and *fftshift()* functions in Matlab, where the former performs the Fourier transform and the latter centres the result, such that the (f_x_,f_y_)=(0,0) component appears at the centre (origin) of the frequency matrix. Note that in the power spectra in Figure 10(d) and 10(e) larger magnitude components are depicted as brighter white, and vice versa. All frequencies were converted to cycles/degree, as described earlier, based on size of the images and the viewing distance of participants.

By combining f_x_ and f_y_ coordinates and expressing frequencies using polar coordinates (Oliva & Torralba, 2001), we defined the *fundamental spatial frequency* as the frequency of the maximum magnitude value from among the peak values along each polar ray emanating from the origin (but excluding points adjacent to the origin), as illustrated by the red dot in Figure 10(e).

Because this metric reveals the spectral power at only a single frequency, however, rather than a *band* of frequencies containing more detailed spectral overtones of any particular pattern, we extended our analysis by operationally defining a new metric, the *Effective Spatial Frequency Bandwidth (ESFB)*. To this end we used the Matlab *findpeaks()* function to identify the set of consecutive peak powers along the radial direction defined by the fundamental frequency, as exemplified by the blue dots in Figure 10(f). Based on a (low-pass) assumption of diminishing peak magnitudes for increasing frequency, we then fit successive peak values along the fundamental radial direction for each pattern using the following exponential decay model:

(3) $$I\left(f\right)≅Ae^{-kf}+C$$

where I(f) is the power spectral magnitude and f is the spatial frequency in polar coordinates.

Because our goal was to categorise and compare visual patterns using the *ordering* of ESFB values, it was deemed sufficient to select an arbitrary, but consistent, threshold value for all patterns. Figure 10(g) illustrates the monotonically decreasing nature of equation (3) and thereby demonstrates why the choice of an arbitrary threshold value (in the present case 1000) within the range of signal values obtained will not affect the *ordering* of the resulting ESFB values. For the patterns used in the experiment, ESFB values in Figure 6(a), for low frequency patterns, ranged up to 8 cycles/deg, whereas for high frequency patterns in Figure 6(b), ESFB ranged up to 23 cycles/deg.

### Participants

Sixty undergraduate engineering students (mean age ∼22, ∼2/3 female) were recruited and after providing informed consent were randomly assigned, 20 each, to the three label conditions. Participants were rewarded with bonus course marks for participation. The study complied with the APA Code of Ethics, and was approved by the Ben-Gurion University ethics committee (Reference #1772-1).

### Hypotheses

All hypotheses were based on the supposition that participants would retain some information about the text-pattern matchings that were encountered during the first phase of the experiment.

**H4**: Both response times (**H4a**) and error rates (**H4b**) were expected to decrease for the stimuli containing patterns (conditions MP and LSFP) relative to stimuli without (condition WL).
**H5**: Patterns comprising lower ESFB values (condition LSFP), with more distinguishable details, were expected to produce reduced response times (**H5**a) and reduced error rates (**H5**b) relative to the higher ESFB patterns (condition MP).

### Results and Analysis

**Figure 11.**
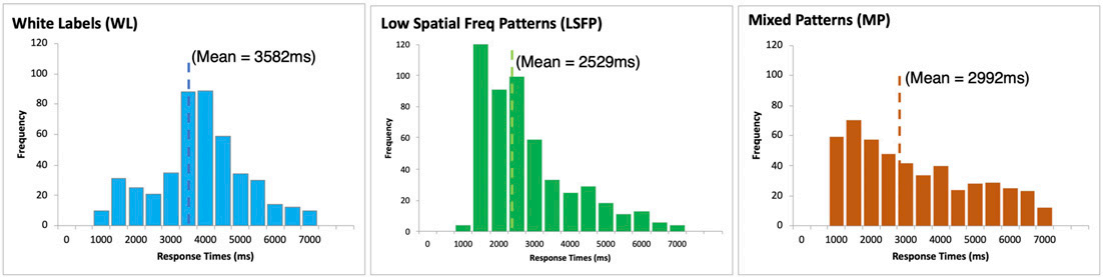
Response time histograms for each condition, including mean values.

To avoid misinterpretation of data due to potential differences in search behaviour during error trials, our RT analysis excluded both error trials and no-response trials (in which targets were not found within the 7s response window), which were analysed separately. A summary of means(+SD) and medians of response times as well as error rates for each dependent variable is presented in Table 3. Figure 11 shows distributions of collective RTs for each condition.

**Table 3.** Mean (+SD) & median response times (RT) and error rates for each condition.

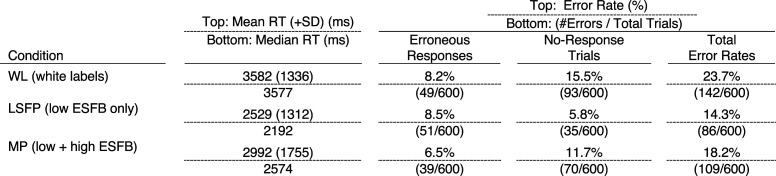

Univariate Anova revealed a significant effect due to conditions (F(2,57) = 20.52, *p* < .0005). Pairwise contrasts revealed that, consistent with Hypotheses H4a and H5a, White Label RTs (mean = 3582 ms) were significantly greater than both LSFP (mean = 2550 ms, *p* < .0005) and MP stimuli (mean = 2992 ms, *p* = .001), and that MP stimuli were significantly greater than LSFP stimuli (*p* = .008).

Univariate analysis of Total Error Rates indicated a significant main effect (F(2,57) = 3.16, *p* = .05), with pairwise contrasts indicating significantly more errors for the WL condition (23.7%) relative only to LSFP (14.3%, *p* = .015). Further analysis of only Erroneous Responses (incorrect choice of target) revealed no significant differences. Analysis of No-Response Trials, on the other hand, showed a significant effect due to conditions (F(2,57) = 7.66, *p* = .001) and post-hoc comparisons revealed that significantly more errors were committed in the WL condition (15.5%) than the LSFP condition (5.8%, *p* < .0005), which was consistent with Hypothesis H4b. The LSFP error rate (5.8%) was also found to be significantly lower (*p* = .023) than for the MP condition (11.7%), which was consistent with Hypothesis H5b.

**Figure 12.**
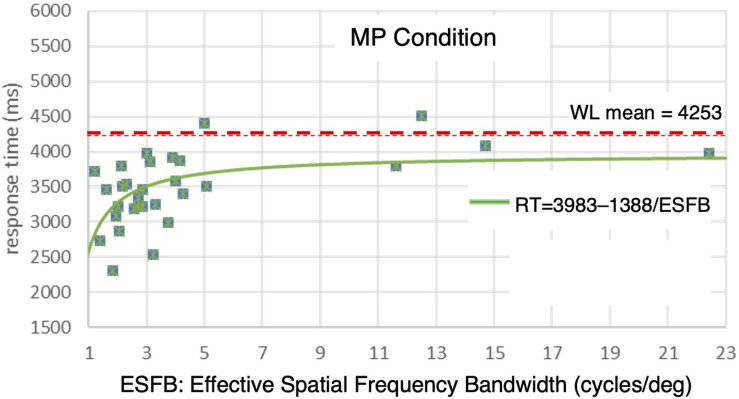
Scatter plot of mean RT vs ESFB values for each target pattern, for MP condition. Note that dashed horizontal line showing WL condition mean does *not* vary with ESFB, but is included for comparative purposes only. Modelling parameters are explained in text.

Figure 12 shows a scatter plot of RT versus ESFB for the MP condition, the one with the larger range of ESFB data and thus most appropriate for examining trends in the data. To characterise these results according to our hypothesis we modelled the data with a function that begins with a minimum RT at very low ESFB (which for mathematical reasons cannot be zero) and for larger values of ESFB increases asymptotically towards a maximum RT value. Our rationale was that as the ESFB of a pattern increases, its details become increasingly more difficult to discern. We thus expect performance to gradually approach the same level as observed with white labels, which contain no background pattern. We have therefore added to the graph, for comparison purposes, a horizontal line showing mean RT from the WL data.

For the sake of parsimony, we modelled our data with the asymptotic function RT=a+b/ESFB . As is evident in Figure 12, the proposed function provides a very convincing fit to the hypothesised behaviour, thereby further supporting Hypothesis H5a in the general sense that response times appear to be smaller for smaller values of ESFB and increase for larger ESFB.

**Figure 13.**
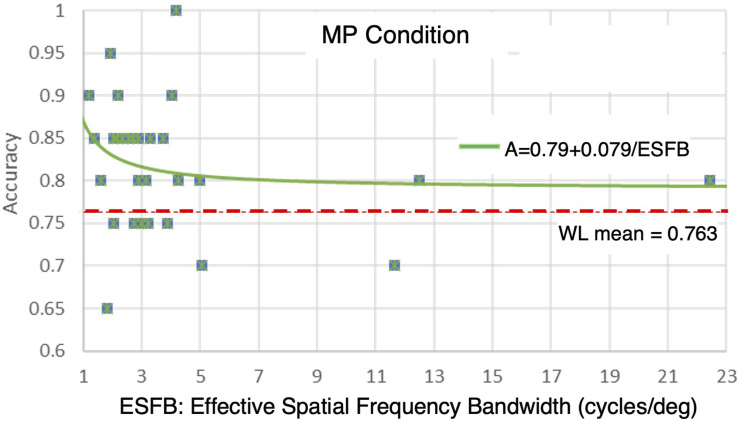
Scatter plot of mean accuracy vs ESFB values for each target pattern, for MP condition. (Dashed lines and model fits explained in Figure 12).

Figure 13 shows analogous results, including asymptotic functions, for the accuracy scores (the converse of the error scores). In accordance with Hypothesis H5b, there is a downward trend in accuracy as spatial frequency increases, with the fitted model once again approaching the mean (minimum) accuracy of the WL condition.

## Discussion

### Summary of Findings

In both experiments, the stimuli comprising only text without a pattern resulted in significantly greater response times than for the patterned stimuli (in support of Hypotheses H1a and H4a). Possibly due to the very low (2–3%) error rates in Study 1, no significant effect of pattern conditions on search accuracy was observed (in contradiction to Hypotheses H2a and H2b). In Study 2, on the other hand, both Hypotheses H4b and H5b were supported, in particular with more overall errors committed in the white label condition. The overall evidence, therefore, does point in the hypothesised direction of search performance being facilitated by provision of supplementary redundant visual patterns.

With regards to whether users are likely to rely on *both* patterns and text in conjunction, rather than processing primarily one of the two modalities, our failure to find evidence of significant differences in both response times (H1b) and accuracy (H2b) for the Pattern-Only versus Text+Pattern conditions in Study 1 prevents us from concluding that the two channels are potentially additive. On the other hand, our finding in Study 2 that significantly more no-response trials (within 7s) occurred for the white label condition relative to the LSFP pattern condition suggests that perhaps the presence of visual patterns reduced the temporal load of reading the stimulus text while searching. Further research on concurrent processing of text and patterns is clearly necessary.

Turning to whether the spatial frequency content of particular patterns may be useful for predicting label effectiveness, our hypothesis was that features in patterns with a lower dominant spatial frequency may be more distinct, thereby potentially supporting guided search, while for higher ESFB levels such features conversely become less distinguishable, resulting in the need for longer, self-terminating search. Only limited RT data (in support of H3a) were obtained in Study 1. However, using a more sophisticated ESFB metric for our Study 2 data resulted in evidence of better performance for lower ESFB patterns, in terms of reduced response times (H5a), and to some extent increased response accuracy (H5b). By modelling those results with an asymptotic function, RTs appeared to increase and accuracy decrease with higher computed ESFB values of the patterns.

### Limitations and Future Research

One potential criticism of our experiments is our use of meaningless or irrelevant text stimuli rather than real-world target words, such as medication names. In Study 1 specifically, it is clear that more meaningful words would have been easier to process than the random consonant strings we used. In Study 2 on the other hand it could be argued that the target words were particularly easy to remember and thus locate. If this were indeed the case, however, we contend that our experimental design would have been biased *against* supporting our hypotheses, in the sense that adding visual patterns should arguably have been less useful when searching for easily identifiable target words. Our findings to the contrary thus plausibly provide even stronger evidence for the potential benefits of added patterns.

In our discussion of Designing for Distinctiveness, we outlined the general concept of guided search theory with regards to its potential use for designing distinctive patterns. In that same section we also speculated that the theory might nevertheless be inadequate for designing *large numbers* of distinctive patterns. Clearly this approach merits further examination.

With regards to the challenge of designing appropriate patterns, we have introduced the concept of an Effective Spatial Frequency Bandwidth (ESFB) as a potential metric for predicting distinguishability. We note, however, that alternative, possibly more effective metrics exist and deserve to be explored. One alternative approach that merits further consideration is Principal Components Analysis of Fourier transformed images (Oliva & Torralba, 2001). Two other potentially useful approaches for characterising image distinctiveness are by quantifying *visual clutter* (Flittner et al., 2020) and *visual crowding* (Pelli et al., 2016). Unfortunately, the majority of literature related to quantifying images is geared towards teaching computer vision systems to understand images, and are thus not necessarily based on the same perceptual criteria used by humans.

In addition to distinguishability, the other important requirement for successful use of supplementary label patterns is that they be *memorable*, where the concept of memorability has been proposed (Bainbridge, 2019) as ‘an intrinsic, measurable property of a stimulus, measuring the likelihood for any given person to remember that image later’. In the present context we have postulated that through repeated exposure over time users will naturally build up a mental model of appearance of those patterns. This aspect of our proposal was not addressed in Study 1, for which the search task relied solely on working memory. In Study 2, some effort was invested towards exposing participants to the patterns that were eventually presented in the data gathering phase. Although ideally each pattern would have been designed according to principles derived from research on memorability, much of that research has focussed on memory of faces, and to some extent scenes (Bainbridge, 2019). More recent research on stimulus-intrinsic properties that influence the memorability of objects, in addition to faces, has revealed that memorability benefit is greater for images that are more efficiently encoded and maintained within visual working memory (Gillies et al., 2023). With increasing relevance to domains such as cognitive science and computer vision, furthermore, it is conceivable that memorability research may eventually provide useful guidelines for the design of effective visual patterns for labelling.

With regards to the stimulus acquainting phase of Study 2, our original intention was that participants would return the next day for the second stage of the preliminary stage, and two days later for the second phase. Unfortunately, due to a variety of constraints experienced by our student participants, the respective time gaps were, as reported above, respectively ‘1–2 days later’ and ‘not sooner than two days after the end of the first phase’. In addition to this shortcoming, which may have negatively affected the efficacy of our procedure, we note also the absence of a control condition that excluded preliminary exposure to the patterns, which also limited our conclusions about the effectiveness of our two-stage implicit learning phase. Further research, preferably longitudinal, is thus necessary to test the memorability of individual patterns.

Despite the above, we nevertheless contend that the significant results reported here can in fact arguably be interpreted as being *conservatively biased*, in the sense that performance improvements such as those observed in our brief exposure experiments, with limited opportunity for getting acquainted with the patterns, would likely be expected to be *even greater* for users who have become well acquainted with their pattern labels through habitual use over time.

Finally, the finding that adding patterns to simple text-based labels significantly improved response times, and to some extent accuracy (and also did not appear to have any negative consequences) suggests that analogous modifications to labelling in real-world applications may reduce both search times and errors due to misidentification. Clearly, more related research is necessary to corroborate this assertion. In particular, the underlying assumption of memorability – that practitioners will over time become familiar with label patterns and thus be able to mentally envisage what they are seeking – is a critical one. On the other hand, it behooves us to recall the criticism of Nunn and Baird (1996) cited earlier, who speculated that using coloured labels might lead to a false sense of security. Clearly the same outcome might occur for adding redundant patterns to labels: if users were to become overconfident by relying on patterns when searching, they might neglect to read the labels! This question clearly also needs to be addressed, ideally under real-world conditions.

#### Application to Medication Labelling, and Beyond

As mentioned, our research was originally motivated by the possibility of reducing the frequency of medical errors. It has been argued that preventable adverse drug events are common across the healthcare system (Aspden et al., 2007) with the frequency of such errors in the US variously estimated to represent 2–14% of hospitalised patients (Williams, 2007), of which 20–30% have been due to packaging or labelling confusion (Berman, 2004; Orser, 2000). The consequent absolute number of such errors, together with their potentially severe implications, thus provide sufficient incentive to try to reduce their frequency.

**Figure 14.**
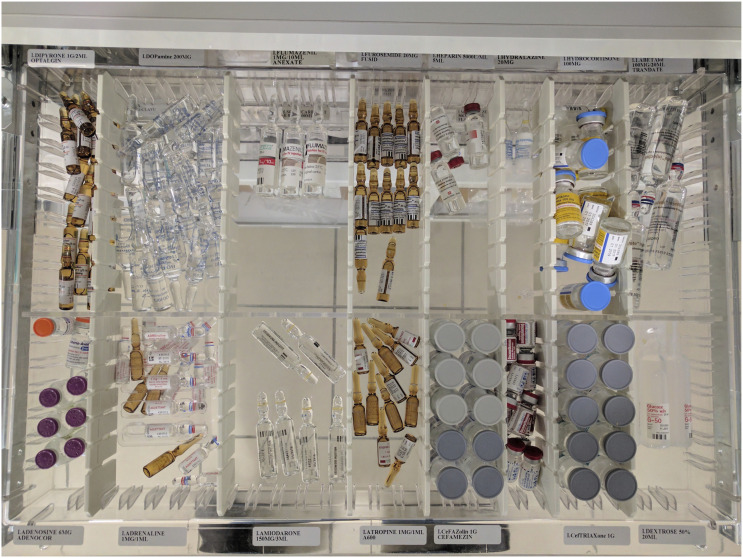
Typical hospital crash cart.

Several examples exist of circumstances associated with the potential for elevated occurrences of errors of substitution in medicine, one of which, illustrated in Figure 14, shows a large variety of medications, many similarly appearing, on a hospital crash cart. It stands to reason that adding distinctive patterns to bottles, vials, ampoules, etc. should cause them to be more easily distinguished from one another, and thus less likely to be selected incorrectly.

**Figure 15.**
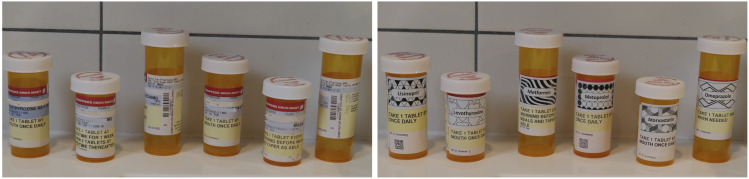
Illustration of patterned label concept on domestic medicine shelf. Left: Simulated home medications, comprising similarly appearing pill containers. Right: Same pill containers, but with visually distinct patterns added to labels.

In Figure 15 we illustrate how our proposed concept might manifest itself on a domestic medicine shelf. On the left we see a situation in which an (elderly) user might require spectacles or supplemental illumination to confirm the identity of a particular medication. On the right, we have added a different distinct pattern (taken from those tested in our study) to each medicine container, as a means of increasing their distinguishability and thereby reducing the likelihood of an incorrect medication being selected.

Returning to the paramedic example described earlier, our premise there is that when administering medication the paramedic not only correctly determines the specific medication and dosage required, but also has a mental image of what that medication looks like. Upon locating it, and prior to administering the medication, she double-checks to confirm that it is the proper medication. The proposed addition of visible patterns, as a supplemental (otherwise unused) information channel, is intended to increase the probability that the paramedic will quickly and successfully reach the correct location of the desired medication.

One might argue that double-checking should negate the need for additional precautions, on the presumption that checks are performed correctly and that any errors encountered are always detected. This is not always the case, however; experienced practitioners have been observed (unintentionally) to perform procedures under ‘autopilot’, potentially resulting in lapses in attention (Merry & Webster, 1996). Confirmation bias can also lead to incorrectly confirming medications, even during double- or triple-checking, based on the expectation that the intended medication has correctly been selected. Poor lighting (especially for paramedics in the field) may also contribute to inadequate checking, while stressful situations may lead to checks being skipped altogether, as a consequence of a perceived lack of time. Finally, the presence of look-alike (and possibly sound-alike) medications can further increase the difficulty of accurately performing checks.

A final important caveat is that our proposed labelling modification may not necessarily be effective for practitioners, such as pharmacists, who manage large numbers of different medications. In other words, whereas it is reasonable to expect that experienced users will be able to learn, through experience, the appearance of *dozens* of distinct patterns, it may not be practical to have similar expectations for remembering *hundreds* of patterns. An additional challenge in this regard would involve the need to design hundreds of maximally distinctive and memorable patterns. Although it is not inconceivable that certain *subsets* of added patterns might be readily recallable and thus provide an advantage for situations involving large numbers of medications, we nevertheless anticipate that potential benefits of this research when applied to medication labelling may be limited to specialities such as paramedicine, anaesthesiology, and emergency medicine, in addition to ordinary consumers, for whom the number of different medications encountered is relatively limited.

Going beyond the anticipated advantages of applying redundant visual labels to medications, this concept is already quite prevalent elsewhere. Various consumer goods that require one to distinguish different flavours within the same brand (such as soft drinks, toothpaste, spice jars, flavoured tea bags, and coffee varieties) as well as industrial equipment like network cables and electrical wires, all employ feature variations to enhance distinguishability. Most such products employ features with a limited range of variations, however, such as colour, size, and orientation. We note with interest here the commercial availability of multi-coloured wrench sets, a product that is consistent with the illustrative example given at the beginning of this paper, albeit without explicit patterns. Our proposed use of maximally distinctive and memorable visual patterns for such applications offers the potential for improved performance (involving reductions in search times, error frequency and beyond) with many such products, particularly in domains such as medication management that encompass a large diversity of items, and especially for products that rely predominantly on colour coding, for which our ability to categorise more than a few colours is limited and with respect to which important guidelines also exist, as a means of accommodating users with colour vision deficiency, to avoid the use of colour as a primary mechanism for conveying information (Campbell et al., 2024).

## Key Points

- It was postulated that adding ‘appropriately designed’ redundant visual patterns to existing labels has the potential to reduce errors of substitution when selecting items (such as medications) from within an ensemble of potentially similarly appearing items.
- The concept of ‘appropriately designed’ is based on patterns that are maximally distinguishable from each other and maximally memorable. In addition to testing the effectiveness of such patterns, suitable tools are required for objectively designing them, including models for predicting effectiveness during the design phase.
- Both studies reported comprised a visual search task, where targets were either Text-Only, Pattern-Only, or Text+Pattern stimuli. Following brief presentation of a target for each trial, participants were required to locate that target from within an array of comparable distractors. Study 1 focussed only on distinguishability, while Study 2 provided additional advance exposure to target stimuli, to enable increased memorability. Results supported hypothesised reduced search times for labels containing patterns, as well as reduced error rates.
- To address the need for objective tools for designing patterns, an ‘Effective Spatial Frequency Bandwidth’ (ESFB) metric was proposed, for which initial results supported the hypothesis that, within constraints of visual acuity, images comprising lower ESFB values are more likely to enhance visual search performance.
